# Are We on Our Way to Achieving the 2020 Goals for Schistosomiasis Morbidity Control Using Current World Health Organization Guidelines?

**DOI:** 10.1093/cid/ciy001

**Published:** 2018-06-01

**Authors:** Jaspreet Toor, Ramzi Alsallaq, James E Truscott, Hugo C Turner, Marleen Werkman, David Gurarie, Charles H King, Roy M Anderson

**Affiliations:** 1 London Centre for Neglected Tropical Disease Research, Imperial College London, United Kingdom; 2 Department of Infectious Disease Epidemiology, School of Public Health, Faculty of Medicine, St Mary’s Campus, Imperial College London, United Kingdom; 3 Center for Global Health and Diseases and Department of Mathematics, Case Western Reserve University, Cleveland, Ohio; 4 The DeWorm3 Project, Natural History Museum of London, United Kingdom; 5 Oxford University Clinical Research Unit, Wellcome Trust Major Overseas Programme, Ho Chi Minh City, Vietnam; 6 Centre for Tropical Medicine and Global Health, Nuffield Department of Medicine, University of Oxford, United Kingdom

**Keywords:** Schistosomiasis, WHO guidelines, morbidity control, elimination as a public health problem

## Abstract

**Background:**

Schistosomiasis remains an endemic parasitic disease affecting millions of people around the world. The World Health Organization (WHO) has set goals of controlling morbidity to be reached by 2020, along with elimination as a public health problem in certain regions by 2025. Mathematical models of parasite transmission and treatment impact have been developed to assist in controlling the morbidity caused by schistosomiasis. These models can inform and guide implementation policy for mass drug administration programs, and help design monitoring and evaluation activities.

**Methods:**

We use these models to predict whether the guidelines set by the WHO are on track for achieving their 2020 goal for the control of morbidity, specifically for *Schistosoma mansoni*. We examine whether programmatic adaptations; namely increases in treatment coverage and/or expansion to adult inclusion in treatment, will improve the likelihood of reaching the WHO goals.

**Results:**

We find that in low-prevalence settings, the goals are likely to be attainable under current WHO guidelines, but in moderate to high-prevalence settings, the goals are less likely to be achieved unless treatment coverage is increased and expanded to at least 85% for school-aged children and 40% for adults.

**Conclusions:**

To improve the likelihood of reaching the WHO goals, programmatic adaptations are required, particularly for moderate- to high-prevalence settings. Furthermore, improvements in adherence to treatment, potential development of candidate vaccines, and enhanced snail control and WASH (water, sanitation, and hygiene) measures will all assist in achieving the goals.

The Neglected Tropical Disease (NTD) Modelling Consortium aims to develop mathematical models of NTD transmission dynamics and the impact of control measures, for infections included in the London Declaration on NTDs [1]. Schistosomiasis was included within this declaration and linked to the World Health Organization (WHO) 2020 roadmap on NTDs. The disease is endemic in 54 countries affecting approximately 240 million people worldwide, with up to 700 million people at risk of infection [2]. Schistosomiasis is an intestinal or urogenital disease caused predominantly by infection with *Schistosoma mansoni*, *Schistosoma haematobium*, or *Schistosoma japonicum*. Individuals become infected when cercariae, released by freshwater snails, penetrate the skin during contact with contaminated water [3]. Due to the aquatic nature of the intermediate snail host, freshwater contact is usually required for an individual to be exposed to infection. However, *Oncomelania* hosts for *S. japonicum* are amphibious, so infection can also occur near contaminated water bodies [4]. Schistosomiasis can result in anemia, chronic pain, diarrhea, and malnutrition, causing poor school performance and lower fitness [5]. The WHO has set recommended guidelines charting routes to the control or elimination of schistosomiasis [6, 7].

Schistosomiasis control has focused on treating populations via mass drug administration (MDA) of praziquantel as a community- or school-based treatment program. As school-aged children (SAC; 5–14 years old) are most likely to be infected by *Schistosoma* parasites, treatment has been specifically focused at this age group. The WHO recommends using prevalence of infection in SAC to determine the treatment frequency (how often to treat) in a given endemic area [2]. The recommended treatment strategy for *Schistosoma* infection is dependent upon whether the community has low, moderate, or high SAC prevalence at baseline before the implementation of preventive chemotherapy (PCT) (Figure 1; baseline prevalence can be calculated in regions where treatment may have been carried out previously [8]). The strategy for low-risk communities is to treat all SAC once every 3 years and treat suspected cases. For moderate-risk communities, the recommendation is to treat all SAC and at-risk adults once every 2 years. For high-risk communities, the recommended approach is to treat all SAC and at-risk adults annually. The WHO guidelines suggest that after 5–6 years of assigned PCT, with a continuously achieved coverage level of >75%, the treatment frequency may be reduced accordingly. If the prevalence remains low for 4 years following a lower treatment frequency, the treatment frequency may be further reduced. Conversely, if the prevalence returns to baseline levels, the previous treatment frequency should be reintroduced [6].

**Figure 1.**
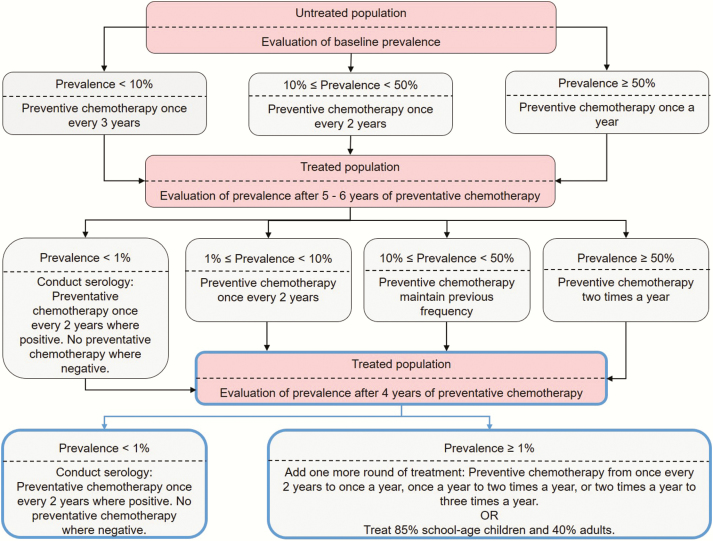
Recommended programmatic adaptations (highlighted in blue boxes) to the current World Health Organization guidelines (in black boxes; using 75% coverage of school-aged children [SAC]) showing the frequency of treatment to be carried out according to prevalence in SAC in the region, where low prevalence is <10%, moderate prevalence is between 10% and 50%, and high prevalence is ≥50% [6].

There is a limited supply and availability of the drug of choice, praziquantel, used to treat infected individuals. Schistosomiasis currently has one of the lowest levels of MDA coverage of all human helminth infections [9, 10]. By 2020, WHO aims to increase coverage such that 75% of SAC at risk will be regularly treated in endemic countries [11] (Supplementary Figure 1). However, young adults (up to 25–30 years old) also make up a large proportion of those infected. Hence, if MDA is only targeted at SAC, a large fraction of the local *Schistosoma* infection burden remains untreated [3]. In 2002, it was recommended that adults be treated in high-risk areas and that women of childbearing age not be excluded from MDA coverage [12]. However, adult treatment has not been regularly implemented in most MDA programs [2, 3]. Notably, praziquantel also provides a preventive measure to female genital schistosomiasis, reducing the risk of human immunodeficiency virus [13–15].

The WHO 2020 target is “morbidity control” by reducing the prevalence of heavy-intensity infections to ≤5% among SAC. After this goal is reached, the next target for 2025 is “elimination as a public health problem,” meaning that the treated region has reached ≤1% prevalence of heavy-intensity infections among SAC. An additional goal is to interrupt transmission of schistosomiasis in the region of the Americas, the Eastern Mediterranean region, the European region, the South-East Asia region, and in selected countries of the African region by 2025 [2]. It is recommended that countries should initially focus on morbidity control across sentinel sites, after which they should focus on elimination as a public health problem in all sentinel sites. Following this, countries may then shift to elimination (interruption of transmission) until the incidence of new infections is reduced to zero. This study examines whether we are on track for reaching the goals recommended by WHO using their current guidelines. Where these guidelines do not appear to be sufficient, we discuss programmatic improvements that could be made to achieve the goals.

## METHODS

In our simulations of PCT impact on the control of schistosomiasis, we followed the WHO-recommended guidelines [6] (Figure 1) using deterministic models developed independently by Imperial College London (ICL) and Case Western Reserve University (CWRU). Beginning with an untreated population, we treated the population for 6 years, with the treatment frequency determined by the baseline prevalence. At year 6, the treatment frequency was reevaluated depending on the prevalence in SAC. The new strategy was then carried out for a further 4 years. We considered scenarios falling within the different treatment frequencies—that is, SAC baseline prevalences <10% (low-prevalence settings), between 10% and 50% (moderate-prevalence settings), and ≥50% (high-prevalence settings) [6]. The intrinsic intensity of transmission (ie, basic reproductive number [R_0_]) [3], was varied in the ICL model, and the transmission coefficients to humans and snails (ie, index of transmission potential [ITP]) was varied in the CWRU model to simulate a range of baseline prevalence levels.

Throughout the 10 years of treatment, we assumed PCT coverage of 75% of SAC only (level of SAC receiving treatment; assumed to be delivered at random at each round within the SAC population). At year 10, the endpoint SAC heavy-intensity infection prevalence was evaluated to determine whether the WHO morbidity and/or elimination as a public health problem goal had been met. Where the goals had not been achieved, we investigated the impact of prolonging treatment or changing to different treatment strategies for 6 additional years starting at year 10. This included the programmatic adaptations of carrying out PCT at the same frequency, treating at a higher coverage level (increasing SAC coverage and/or inclusion of adults), or increasing treatment frequency. Our models simulated what happened as the WHO guidelines were followed for various scenarios while projecting both the prevalence of infection (eggs per gram [epg] >0) and prevalence of heavy-intensity infections (epg ≥400 [2, 7]) in SAC throughout the treatment period, acknowledging that there may be individuals with heavy-intensity infections in any prevalence setting. See Supplementary Table 1 for parameter values used within the models; here we focus on *S. mansoni*.

## RESULTS

**Figure 2.**
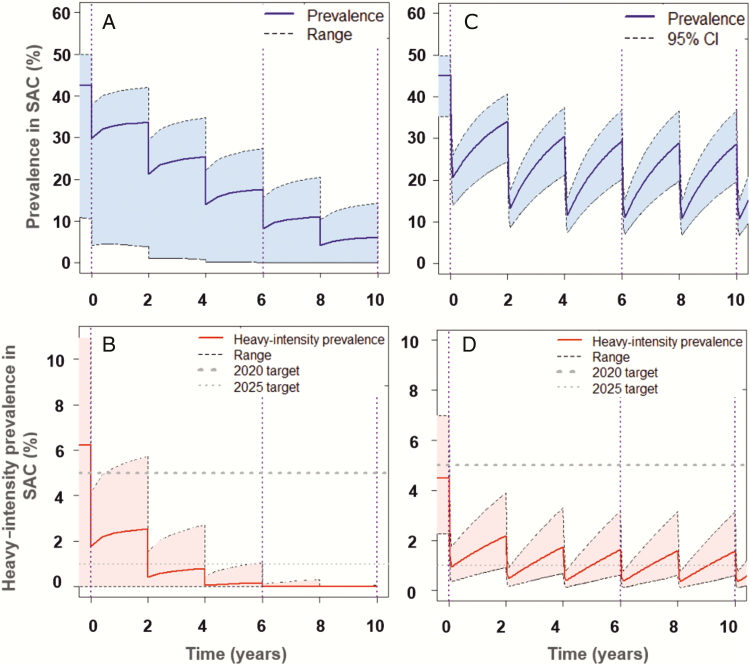
Imperial College London (*A* and *B*) and Case Western Reserve University (CWRU) (*C* and *D*) model scenarios showing prevalence of infection (eggs per gram [epg] >0) (*A* and *C*), and prevalence of heavy-intensity infections (epg ≥400) (*B* and *D*), in school-aged children (SAC) for settings of moderate baseline prevalence. Preventive chemotherapy once every 2 years reaches the morbidity goal by year 6 and may reach the elimination as a public health problem goal by year 10 (reached in 20% of the CWRU simulations). *A* and *B*, Shaded areas represent the range of basic reproductive number (R_0_) values (R_0_ = 1.22–1.241). *C* and *D*, Shaded areas represent the 95% confidence interval (CI) of uncertainty with the range of index of transmission potential (ITP) values (ITP = 1–5.6). The corresponding projections for the incidence of infection in the population are shown in Supplementary Figure 12*B*.

**Figure 3.**
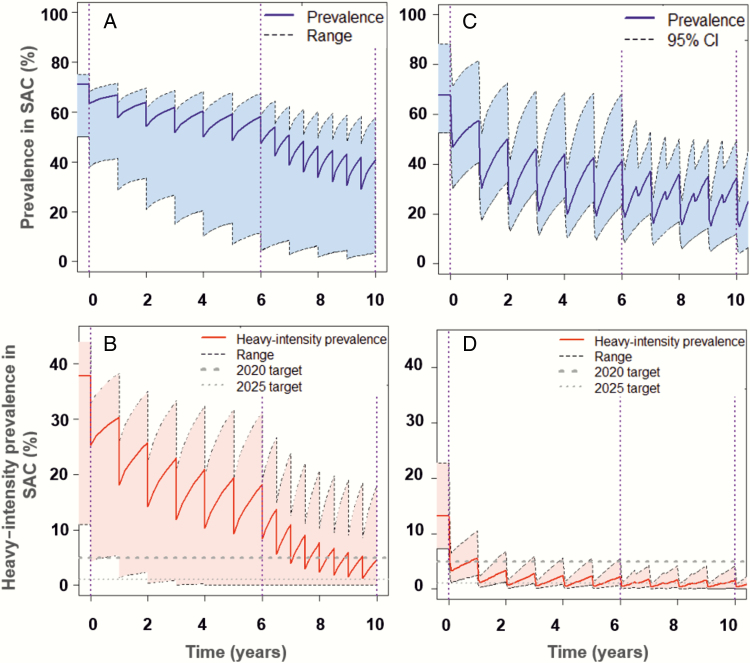
Imperial College London (*A* and *B*) and Case Western Reserve University (CWRU) (*C* and *D*) model scenarios showing prevalence of infection (eggs per gram [epg] >0) (*A* and *C*), and prevalence of heavy-intensity infections (epg ≥400) (*B* and *D*), in school-aged children (SAC) for settings of high baseline prevalence. Preventive chemotherapy (PCT) is carried out once a year for 6 years and then at the same frequency or twice a year for 4 years depending on year 6 prevalence, which may reach one, both, or no goals (elimination as a public health problem goal reached by year 10 in 36% of the CWRU simulations). *A* and *B*, Shaded areas represent the range of basic reproductive number (R_0_) values (R_0_ = 1.2421–5.0). *C* and *D*, Shaded areas represent the 95% confidence interval (CI) of uncertainty with the range of index of transmission potential (ITP) values (ITP = 1–5.6); jagged lines due to 19% of the simulations having prevalence ≥50% at year 6 and therefore subjected to twice-yearly PCT. The corresponding projections for the incidence of infection in the population are shown in Supplementary Figure 12*C*.

Following the WHO-recommended treatment coverage for SAC at 75%, it is likely that the elimination as a public health problem goal will be achieved in low-prevalence regions within 6 years of treatment (Supplementary Figure 2). This finding aligns with previous publications [9]. For moderate-prevalence regions, the morbidity goal is achieved within 6 years of treatment, but the elimination goal may or may not be achieved within 10 years (Figure 2). Similarly, following 10 years of treatment, the WHO guidelines are unlikely to achieve either goals in high-prevalence regions (Figure 3). In moderate- to high-prevalence settings where one or both goals are not attainable following 10 years of PCT, further intervention strategies would be necessary, such as increased treatment frequency and/or increased treatment coverage with expansion to include adult treatment.

The scenarios where WHO goals were not met were reevaluated to determine which improvements could be implemented. These programmatic adaptations were simulated for an additional 6 years, continuing from year 10. First, we tested continuation of the decision 2 treatment frequency (Supplementary Figures 3 and 4). Second, we tested continuation of the decision 2 treatment frequency at increased coverage, that is, inclusion of adult coverage at 40% and/or increased SAC coverage at 85% (Supplementary Figures 5–9). The results for both options predicted some improvement as the WHO goals were reached in more of the simulations. Of greatest importance was the result that the inclusion of adult treatment and higher SAC coverage led to a higher probability of achieving the elimination as a public health problem goal; the probability of achieving this increased to 24% and 48% (from 21% and 42%) for moderate- and high-prevalence settings, respectively (Supplementary Figures 8 and 9).

In a third approach, when PCT was adapted by increasing the treatment frequency for moderate-prevalence settings at year 10, from once every 2 years to annual, the probability of achieving the elimination as a public health problem goal increased to 81% at year 16. Additionally, increasing the treatment frequency for high-prevalence settings at year 10, from annually to twice a year, or from twice a year to 3 times a year, the elimination as a public health problem goal may be achieved, with the probability of achieving this increasing to 78% (Supplementary Figures 10 and 11). However, this third approach is not advisable due to logistical reasons such as treatment adherence [3, 16–19].

It is important to note that typically the SAC prevalence of infection and incidence were still high, even in cases where the SAC prevalence of heavy-intensity infections reached the WHO goals (Figures 2 and 3; Supplementary Figures 2, 12, and 13). Hence, transmission was maintained and treatment would have to continue indefinitely unless treatment is shifted to a transmission elimination strategy. This requires at least community-wide treatment, especially in moderate- to high-prevalence regions.

Comparing the results in Supplementary Tables 2 and 3, we see that for our range of ITP and R_0_ values, the CWRU model predicts that the morbidity goal is likely to be reached in all prevalence settings, whereas the ICL model shows this is unlikely in high-prevalence settings. This is due to differing model assumptions (Supplementary Data and [3, 20]). Despite these differences, both models generally agree on whether the WHO goals will be achieved using the recommended guidelines. In low-prevalence regions, both goals are likely to be achieved; in moderate-prevalence regions, the morbidity goal is achieved with the elimination goal possibly being achieved; in high-prevalence regions, one, both, or neither of the goals are likely to be achieved.

## OTHER INTERVENTIONS

### Expansion From School-Based to Community-wide Treatment Programs

School-based treatment programs are widely used to target SAC for schistosome infection control. An alternative is to use community-wide MDA to target the whole community as has been employed for other human helminth infections [21]. Community-wide treatment, through a variety of delivery platforms [22, 23], can be more effective and cost beneficial for controlling schistosomiasis transmission than school-based treatment [24]. However, in terms of morbidity control, the benefit of community-wide treatment is highly variable [24].

To achieve morbidity control in SAC, the best strategy may be to scale up the geographical coverage of the school-based programs (which is currently low in many settings) and prioritize community-wide treatment in settings where the adult burden and transmission intensity are known to be high, as well as in settings where school enrollment or coverage is low [24].

Although 250 million praziquantel tablets are expected to be donated in 2017, this is only sufficient to treat SAC in need [25], leaving the global coverage of adults at only 14.3% [26]. In areas requiring adult coverage, more funding and donations are needed for adult treatment, along with an expansion of the global availability of praziquantel [24]. For programs requiring community-wide treatment, integration of schistosomiasis treatment with other NTD interventions will allow programs to capitalize on existing infrastructures, capturing economies of scope [7, 27].

### Treatment Adherence

Although in our projections we assume 75% SAC coverage, this may not be “real” coverage. A proportion of the eligible population may be persistent nonadherers, not taking treatment at any round [3, 16, 18, 19, 28]. A variety of factors can result in nonadherence, such as treatment accessibility, dropout from treatment programs, relationships between drug distributers and the local population, and unawareness of the disease causes or the benefits of MDA [18, 28, 29]. This hinders fast scale-up of treatment coverage and reduces MDA impact.

It is important to assess whether treatment programs should focus specifically on reaching nonadherers or on increasing overall coverage. To prevent local reinfections, infected nonadherers need to be treated as they create a reservoir of infection by continuing to carry the infection and releasing infective stages into the environment [17]. More adherence data should be a priority as this would inform the real coverage being achieved in treatment areas.

### Potential Vaccine, Snail Control, and WASH

Our models predict that the current WHO guidelines, with praziquantel as the backbone, will fall short of interrupting transmission, especially in moderate- to high-prevalence settings within a practicable time (6–10 years). As treatment with praziquantel does not reduce the possibility of reinfection, additional interventions, such as development of an effective vaccine [30, 31] or implementation of carefully timed snail control measures [32], are required to generate a rapid but long-lasting decline in incidence and prevalence that would make elimination feasible within a manageable time frame (eg, a decade).

Other interventions include improving water, sanitation, and hygiene (WASH) through the provision of clean water plus sanitation and reduction of water contact [33, 34]. Although these interventions reduce transmission, the lack of good data on their efficacy limits model predictions of their potential impact.

## DISCUSSION

In this study, we have investigated whether we are on track for reaching the WHO goals by following their current recommended guidelines [6]. Where the WHO goals were not achieved, we explored programmatic adaptations that could be implemented to increase the likelihood of reaching these goals.

The goals of morbidity control and elimination as a public health problem have been set by WHO to be reached by 2020 and 2025, respectively. We found that the likelihood of achieving the goals varies depending on the baseline prevalence (and hence intrinsic transmission potential) in a region. In low-prevalence regions, the current WHO guidelines with 75% SAC coverage are likely to achieve elimination as a public health problem within 6 years. In moderate-prevalence regions, the morbidity goal could be reached within 6 years and the elimination goal may be reached within 10 years depending on the transmission potential in the region. However, the current guidelines have a low likelihood to meet the goals in high-prevalence regions. These results are summarized in Table 1 and incorporated in Figure 1.

**Table 1.** Summary of Model Projections After Following the Recommended Guidelines Set by the World Health Organization (WHO) and Suggestions for Programmatic Adaptations in Cases Where the WHO Goals Are Not Achieved for *Schistosoma mansoni*

Baseline Prevalence in SAC	Morbidity Goal Reached?	Elimination as a Public Health Problem Goal Reached?	Programmatic Adaptation
Low (<10%)	Yes; within 6 years	Yes; within 6 years	Not required
Moderate (10%–50%)	Yes; within 6 years	Varies depending on scenario	Increase PCT to once a year OR increase coverage to 85% for SAC and include adult treatment at 40% coverage
High (≥50%)	Varies depending on scenario	Varies depending on scenario	Increase PCT from once a year to twice a year (where year 6 prevalence has fallen between 10% and 50%) or from twice a year to 3 times a year (where year 6 prevalence has fallen ≥50%) OR increase coverage to 85% for SAC and include adult treatment at 40% coverage^a^

The World Health Organization goals are shown in Figure 1.

Abbreviations: PCT, preventive chemotherapy; SAC, school-aged children.

^a^We recommend increasing coverage and expanding to include adult coverage rather than increasing treatment frequency to 2 or 3 times a year due to logistical issues, such as adherence to treatment.

In moderate- to high-prevalence settings, where the guidelines do not reach the WHO goals, there are programmatic improvements in coverage and/or frequency of treatment that should be made. By adapting the WHO guidelines to include adult treatment with a coverage of at least 40% and/or increased coverage of SAC at ≥85%, the WHO goals are more likely to be attainable. Alternatively, increasing the treatment frequency also yields a higher chance of achieving elimination as a public health problem within a shorter time span, especially in areas of high intrinsic transmission potential. Broadening and deepening coverage (though difficult), rather than increasing treatment frequency, would be beneficial in logistical terms due to issues such as treatment adherence [3, 16–19].

Our results varied depending on the age-intensity profile of infection in a region. The models predict that in regions where SAC carry the majority of the infection, the WHO goals become harder to reach as they are defined by heavy-intensity infection prevalence in SAC only and this prevalence is correspondingly higher when SAC have a greater burden of infection. In this case, a higher treatment frequency or higher SAC coverage level, and/or community-wide treatment, are needed to improve the likelihood of reaching the WHO goals. Similarly, in settings with higher transmission potentials than those used within our model (and in persistent hotspots [35]), the likelihood of achieving the WHO goals and of interrupting transmission decreases.

Other interventions, such as improving WASH, the availability of a vaccine, and the incorporation of snail control, could be beneficial in addition to praziquantel-based MDA to assist in achieving the WHO goals. However, despite promising results of some candidates in animal models, it is important to note that a vaccine is unlikely to become available before 2025. Targeting persistent nonadherers for treatment is also important as such individuals result in the WHO goals being less likely to be reached.

Current microscopic diagnostic techniques for schistosome infection, such as Kato-Katz, have measurement errors, particularly at low prevalence levels. Using diagnostic techniques with increased sensitivity is increasingly important as we move toward elimination. A relatively newer diagnostic technique, point-of-care circulating cathodic antigen [36], will assist in improving accuracy as we look toward elimination as a public health problem and further on toward transmission elimination.

Although we have shown that the prevalence of heavy-intensity infections is reduced by adhering to the WHO guidelines, prevalence of infection and incidence may still be high, meaning transmission will not have been significantly reduced and hence treatment will have to be continued indefinitely. Reevaluation is required on whether the WHO should aim to reduce prevalence of infection in addition to the prevalence of heavy-intensity infections, particularly in endemic regions where treatment programs have been active for many years. To achieve transmission elimination, an increase and expansion across age classes in coverage levels and/or frequency of treatment is required.

We have focused on *S. mansoni* but our models can be parameterized for *S. haematobium*. There is uncertainty in certain model parameter values, such as the age-specific rates of transmission as defined in the age-intensity profile of infection and the importance of acquired immunity in older age groups. More accurate data are needed to improve our model predictions. Our model results are constrained by the range of R_0_ or ITP values used, and the assumptions made on coverage levels with 100% treatment adherence. Throughout our projections and programmatic adaptations, we have been grounded by the current WHO guidelines, but these could be optimized by altering the low-, moderate-, or high-prevalence thresholds.

## CONCLUSIONS

Using our model-based analyses to investigate whether we are on track for achieving the WHO goals using their current guidelines, we have found that this depends heavily on the transmission setting. In low-prevalence regions, there is a high likelihood of achieving the goals. However, in moderate- to high-prevalence regions, programmatic adaptations are required to make the goals achievable. Modifications to the current guidelines, such as deeper and broader age group treatment coverage levels, will increase the likelihood of achieving the goals. By presenting these results, we hope this study will stimulate discussions on what the future WHO schistosomiasis guidelines should be.

## Supplementary Data

Supplementary materials are available at *Clinical Infectious Diseases* online. Consisting of data provided by the authors to benefit the reader, the posted materials are not copyedited and are the sole responsibility of the authors, so questions or comments should be addressed to the corresponding author.

Supplementary Material

## Notes

***Author contributions.*** J. T. carried out the analysis of the ICL model, produced the results, and wrote the first draft. J. E. T. developed the ICL model code and assisted with the model analysis. R. A. developed the model code, conducted the model analysis, and produced the results for the CWRU model. J. T., H. C. T., M. W., J. E. T., R. A., D. G., C. H. K., and R. M. A. contributed to the writing of the manuscript and the definition of the mathematical models. All authors contributed to discussions on the design of the study.

***Acknowledgments.*** We thank Deirdre Hollingsworth and Luc Coffeng for helpful comments and discussions on the study; Hajnal Farkas for managing the NTD Modelling Consortium; and Susan P. Montgomery (Centers for Disease Control and Prevention [CDC]), W. Evan Secor (CDC), and Pauline N. M. Mwinzi (Centre for Global Health Research, Kenya Medical Research Institute), for sharing data from the Schistosomiasis Consortium for Operational Research (SCORE) Sm2 “Gaining Control” project based in Kenya.

***Financial support.*** This work was supported by the Bill & Melinda Gates Foundation in partnership with the Task Force for Global Health through the NTD Modelling Consortium (grant number OPP1053230) and the Children’s Investment Fund Foundation (UK) to J. T., R. A., D. G., C. H. K., and R. M. A. This work was also supported by the Bill & Melinda Gates Foundation for research grant support via the DeWorm3 (grant number OPP1129535) award to the Natural History Museum in London to J. E. T., M. W., and R. M. A; the Wellcome Trust (grant number 089276/B/09/7 to H. C. T.); and SCORE based at the University of Georgia (to R. A., D. G., and C. H. K.).

***Disclaimer.*** The views, opinions, assumptions, or any other information set out in this article are solely those of the authors and should not be attributed to the funders or any person connected with the funders. GlaxoSmithKline (R. M. A.) played no role in study design, data collection and analysis, decision to publish, or preparation of the manuscript.

***Supplement sponsorship.*** This article appears as part of the supplement “Reaching the 2020 Goals for Nine Neglected Tropical Diseases,” sponsored by the NTD Modelling Consortium.

***Potential conflicts of interest.*** R. M. A. is a nonexecutive director of GlaxoSmithKline. All other authors report no potential conflicts. All authors have submitted the ICMJE Form for Disclosure of Potential Conflicts of Interest. Conflicts that the editors consider relevant to the content of the manuscript have been disclosed.
