# Supplementary material for: Are We on Our Way to Achieving the 2020 Goals for Schistosomiasis Morbidity Control Using Current World Health Organization Guidelines?
Source: Clin Infect Dis. 2018 Jun 1;66(Suppl 4):S245–52. doi: 10.1093/cid/ciy001 (PMC5982704; doi:10.1093/cid/ciy001)
Supplement: Supplementary Material [file ciy001_suppl_supplementary_material.docx]

**Supplementary Information**

**Summary of previous model insights**

As part of the Neglected Tropical Disease (NTD) Modelling Consortium, models of schistosomiasis control have been independently developed and analysed by groups based at Imperial College London (ICL) and Case Western Reserve University (CWRU; with participation from Yale University in the development and calibration of the CWRU model). See [1] for a recent comparison of the ICL and CWRU models and [2] for a broader review of past work on mathematical models of schistosome parasites and their control. Previous work by the two main NTD Modelling Consortium groups has suggested treatment strategies for various transmission settings as highlighted below. Past focus has been on who to treat, how often to treat, how many to treat (coverage of target group), how long to treat to achieve a given target, and how these factors are influenced by the transmission setting.

- ICL model: In low to moderate transmission settings, 75% coverage in SAC and 27.4% coverage in adults through annual rounds of mass drug administration (MDA) is recommended [3]. This reduces mean intensities of infection to very low levels by 2020 and crosses the transmission breakpoint, eliminating transmission, by 2030 [3]. In high transmission settings, higher levels of coverage and the inclusion of adult coverage are essential to interrupting transmission [3].
- CWRU model: In low and high transmission settings, infection prevalence can be reduced within 10 years given high community coverage (> 70%) with at least annual MDA [4]. There is a significant risk of bounce-back if MDA is suspended. Water, sanitation and hygiene (WASH) measures and snail control are important in preventing re-emergence. In high transmission settings, elimination of schistosomiasis, measured by reducing the incidence of infection (new worms acquired per person per year) to zero, would be difficult to reach within the next 30 years by using only annual MDA. Further interventions are needed in addition to MDA to interrupt transmission in these settings [5].

**Model differences**

There are key differences between the ICL and CWRU models. The ICL model is an age-structured, deterministic model which considers the dynamics of the mature adult worm in the human host whilst incorporating a negative binomial distribution of parasites per host (with an aggregation parameter k), density dependence in fecundity and mating probabilities. The ICL model structure collapses the details of the dynamics of the snail host and the two larval stages into the term for the basic reproductive number, R_0_ [6], due to the great differences in life expectancies of the different life-cycle stages (hours for miracidia and cercaria, days to week for infected snails, and 3.5-10.5 years for the adult parasite in humans). This approach is standard for systems of differential or partial differential equations, where equations in the system have orders of magnitude differences in rates of turnover.

The CWRU model is a deterministic model that includes details on the parasite life cycle and dynamics of the intermediate snail host whilst incorporating a stratified worm burden (SWB) approach, such that the host population is divided into classes harbouring different worm burdens. Here, SWB is stratified by 126 strata with a worm burden increment of 2 worms. Prevalence (defined by 1-probability of zero eggs) is calculated according to de Vlas model [7]; hence even high burden strata contribute to zero eggs (as there is a non-vanishing probability for them to do so). By incorporating an explicit handling of the transmission to snails, the CWRU model separates factors that affect human prevalence levels from those likely having indirect effects on transmission, such as the parameters controlling the force of infection to snails. The index of transmission potential (ITP) in the CWRU model is a function of several parameters among which are the transmission coefficients from snails to humans and humans to snails. Prevalence is highly sensitive to the transmission coefficients (human-to-snail and snail-to-human). The product of these two coefficients is proportional to the magnitude of R_0_.

In both models, the R_0_ values or the ITP in the life-cycle are specified by parameter estimation procedures based on epidemiological data, especially that on infection in the human host (the age intensity and age prevalence profiles). Differences in model predictions arise from the assumptions made about the worm distribution per host since this distribution determines the numbers in the high intensity classes (negative binomial in the ICL model and a more homogenous distribution of worms amongst hosts in the CWRU model) [1]. In both models, the human infectivity is a function of the mean worm burden. The rate of infection of snails is directly proportional to the product of the density of susceptible snails times the density of miracidia. This assumption is supported by a set of experiments that varied snail and miracidial densities [8, 9]. A general treatment of differences in model structures is given in [1] and [2].

**Model code**

The code for the ICL and CWRU deterministic models have been made available by the NTD modelling consortium [10]. The results can be produced for this paper using the parameter values specified in **Table S1** and by setting the treatment frequency, coverage and length of treatment accordingly.

**Uncertainty and sensitivity to parameters**

Our results are sensitive to the R_0_ and ITP values used within the ICL and CWRU models, respectively; a range of values were used for these parameters to simulate regions falling within the various baseline prevalence categories (shown throughout our results). There is uncertainty in the results due to a lack of data on multiple parameter values. Different age specific contact rates will produce different results as regions with a higher burden of infection in SAC are more likely to fail in achieving the WHO goals. Here we have used parameter values fitted to Iietune village data [2] and SCORE Sm2 ‘Gaining Control’ project data [11] for the ICL and CWRU models, respectively. This analysis could be applied to other specific region settings.

Model predictions are also sensitive to the precise biological assumptions made (structural sensitivity). Both models assume the convex shape of the age-intensity of infection profiles is the result of age related exposure to infection. Acquired immunity is not represented in the model structures. Evidence for the relative roles of age related exposure and the buildup of acquired immunity is very limited, despite much study over many decades [8]. Further research on model development with both age related exposure and the buildup of acquired immunity is a priority for future work. Other aspects for future research on model development is the inclusion of spatial structure in transmission given the focal nature of schistome infection in endemic regions.

We have focused on parameter values for *S. mansoni* but this work could be extended to *S. haematobium*. Furthermore, the ICL stochastic code could be used to look further towards transmission elimination and analyse whether this can be achieved in a defined region with a specified R_0_ value.

**Table S1:** Parameter values used in the ICL and CWRU model for *S. mansoni.*

| **Parameter** | **ICL value** | **CWRU value** | **Value references** |
| --- | --- | --- | --- |
| Fecundity (λ) | 0.34 eggs/female/sample | 1.33; (0.89 - 1.79)^3^ eggs/female/42mg-sample | ICL: [7, 12, 13]  CWRU: [11] |
| Egg distribution within the individual (k_epg_) | 0.87 | 0.17; (0.12 - 0.44)^3^ | ICL: [7, 12]  CWRU: [11] |
| Coefficient of variation | 0 - 2.04^1^ | 2.9; (2.2 - 3.7)^2, 3^ | ICL: [14, 15]  CWRU: [11] |
| Worm lifespan (years) | 5.7 | 5.7 | ICL & CWRU: [13, 16] |
| Drug efficacy (%) | 86.3 | 86.3 | ICL & CWRU: [17] |
| R_0_ values (for ICL model) or index of transmission potential (ITP) values (for CWRU model) | 1.19 – 5.0 | 1.0 - 5.6 | - |
| Age specific contact rates | For 0-4, 5-9, 10-15, 16+ years of age^4^:  0.032, 0.162, 1, 0.06 | For 0-4, 5-14, 15+ years of age^3^:  0.38, 1, 0.02 | ICL: [2]  CWRU: [11] |
| SAC prevalence (%) | SAC having egg count threshold > 0 | SAC having eggs/gram (epg) > 0 | - |
| SAC Heavy-intensity infection prevalence (%) | SAC having egg count threshold > 16 | SAC having epg ≥ 400 | ICL: [18] |

^1^ This value is approximated for female worms in SAC and varies with the R_0_ value. This value is calculated by the ratio of the square root of the variance to the mean number of worms in SAC, where variance is given by (SAC mean worm burden/k) x (SAC mean worm burden + k); here aggregation parameter k=0.04 for low SAC baseline prevalence settings and k=0.24 for high SAC baseline prevalence settings.

2 CWRU model does not use binomial distribution for worms. This value is calculated by the ratio of the standard deviation to the mean of the equilibrium distribution of the number of worms over the stratified worm burden strata.

^3^ Parameters calibrated to reflect data from the SCORE Sm2 ‘Gaining Control’ project based in Kenya for the high prevalence setting [11]. ^4^ Parameters fitted to data from Iietune, Kenya [2].

**Table S2:** Projected outcomes from ICL model for *S. mansoni* according to WHO guidelines shown in **Figure 1**. Results for different baseline prevalences produced by varying R_0_ within the model. Prevalence and heavy-intensity prevalence for SAC are shown at baseline, midpoint (6 years) and endpoint (10 years). R_0_ values shown in black correspond to the lower and upper ranges and those in red correspond to the solid lines plotted in **Figures S2a-b, 2a-b, 3a-b**.

|  |  | **1^st^ decision (for 6 years):** | | |
| --- | --- | --- | --- | --- |
| **2^nd^ decision (for 4 years):** |  | **Prevalence < 10%**  **PCT once every 3 years** | **10% ≤ Prevalence < 50%**  **PCT once every 2 years** | **Prevalence ≥ 50%**  **PCT once a year** |
|  | **Prevalence < 10%**  **PCT once every 2 years** | Prevalence:  8.4–9.8% 🡪 4.1-5.5% 🡪 0.04-0.2%  Heavy-intensity prevalence:  0.7-1.2% 🡪 0.04-0.1% 🡪 0%  R_0_ = 1.205 - 1.215 (1.21) |  |  |
|  | **10% ≤ Prevalence < 50%**  **PCT maintains previous frequency** |  | Prevalence:  10.1-49.9%🡪  4.1-27.5%🡪0.1-14.3%  Heavy-intensity prevalence:  1.3-10.9%🡪0.03-1.1%🡪  0-0.1%  R_0_ = 1.22 - 1.24 (1.19) | Prevalence:  50-67.8%🡪11.5-48.3%🡪3.3-40.4%  Heavy-intensity prevalence:  11-32.1%🡪0.01-9%🡪0-4.7%  R_0_ = 1.24 - 2.3 |
|  |  |  |  | Prevalence:  68.1-68.4%🡪49.1-49.9%🡪41.7-42.9%  Heavy-intensity prevalence:  32.5-33% 🡪 9.6-10.2% 🡪 5.2-5.8%  R_0_ = 2.35 - 2.4 |
|  | **Prevalence ≥ 50%**  **PCT twice a year** |  |  | Prevalence:  68.6-71.3% 🡪 50.7-58.4%🡪 28.5-41%  Heavy-intensity prevalence:  33.4- 37.9% 🡪 10.8-18.5% 🡪1.1-4.9%    R_0_ = 2.45 - 3.15 (3.12) |
|  |  |  |  | Prevalence:  71.5-75% 🡪 58.9-67.5% 🡪41.8-57.9%  Heavy-intensity prevalence:  38.2-43.9% 🡪 19- 31.4% 🡪 5.2- 17.9%  R_0_ = 3.2 - 5.0 |

Will not reach WHO goals Will reach WHO morbidity goal Will reach WHO morbidity and elimination goals

Will reach WHO morbidity goal and possibly elimination goal

**Table S3:** Projected outcomes from CWRU model for *S. mansoni* according to WHO guidelines shown in **Figure 1**. Results for different baseline prevalences produced by calibrating transmission parameters to reflect prevalence and heavy prevalence in settings of high pre-control prevalence in Kenya and scaling the transmission coefficients to humans and snails within the model to get low and moderate baseline prevalence levels. Prevalence and heavy-intensity prevalence for SAC are shown at baseline, midpoint (6 years) and endpoint (10 years). The index of transmission potential falls within the range 1-5.6 irrespective of the baseline prevalence.

|  |  | **1^st^ decision (for 6 years):** | | |
| --- | --- | --- | --- | --- |
| **2^nd^ decision (for 4 years):** |  | **Prevalence < 10%**  **PCT once every 3 years** | **10% ≤ Prevalence < 50%**  **PCT once every 2 years** | **Prevalence ≥ 50%**  **PCT once a year** |
|  | **Prevalence < 10%**  **PCT once every 2 years** | Prevalence:  8.5 (5.6-10)% 🡪5.3 (3.4-6.2)% 🡪4.0 (2.6-4.8)%  Heavy-intensity prevalence:  0.3 (0.09-0.5)% 🡪 0.15 (0.05-0.3)% 🡪 0.1 (0.03-0.2)%  Probability of elimination by year 6: 100% |  |  |
|  | **10% ≤ Prevalence < 50%**  **PCT maintains previous frequency** |  | Prevalence:  45 (35.2-50.0)% 🡪 29.2 (20.0-36.6)% 🡪 28.4 (19.4-36.4)%  Heavy-intensity prevalence:  4.5 (2.3-7.0)% 🡪 1.6 (0.6-3.1)% 🡪 1.6 (0.6-3.1)%  Probability of morbidity/elimination goals by year 10: 100% / 20% | Prevalence:  65.8 (52.4-82.0)% 🡪 35.8 (23.0-47.3)% 🡪 32.3 (11.5-47.1)%  Heavy-intensity prevalence:  12.0 (7.1-21.4)% 🡪1.8 (0.6-4.2)% 🡪 1.6 (0.2-4.2)%  Probability of morbidity/elimination goals by year 10: 99% / 35% |
|  | **Prevalence ≥ 50%**  **PCT twice a year** |  |  | Prevalence:  79.9 (67.2-96.3)%🡪60.9 (50.5-81.5)%🡪39.1 (29.2-55.2)%  Heavy-intensity prevalence:  17.8 (11.5-25.1)%🡪3.6 (0.8-7.4)%🡪1.2 (0.1-2.9)%  Probability of morbidity/elimination goals by year 10: 100% / 43% |

Will not reach WHO goals Will reach WHO morbidity goal Will reach WHO morbidity and elimination goals

Will reach WHO morbidity goal and possibly elimination goal

**
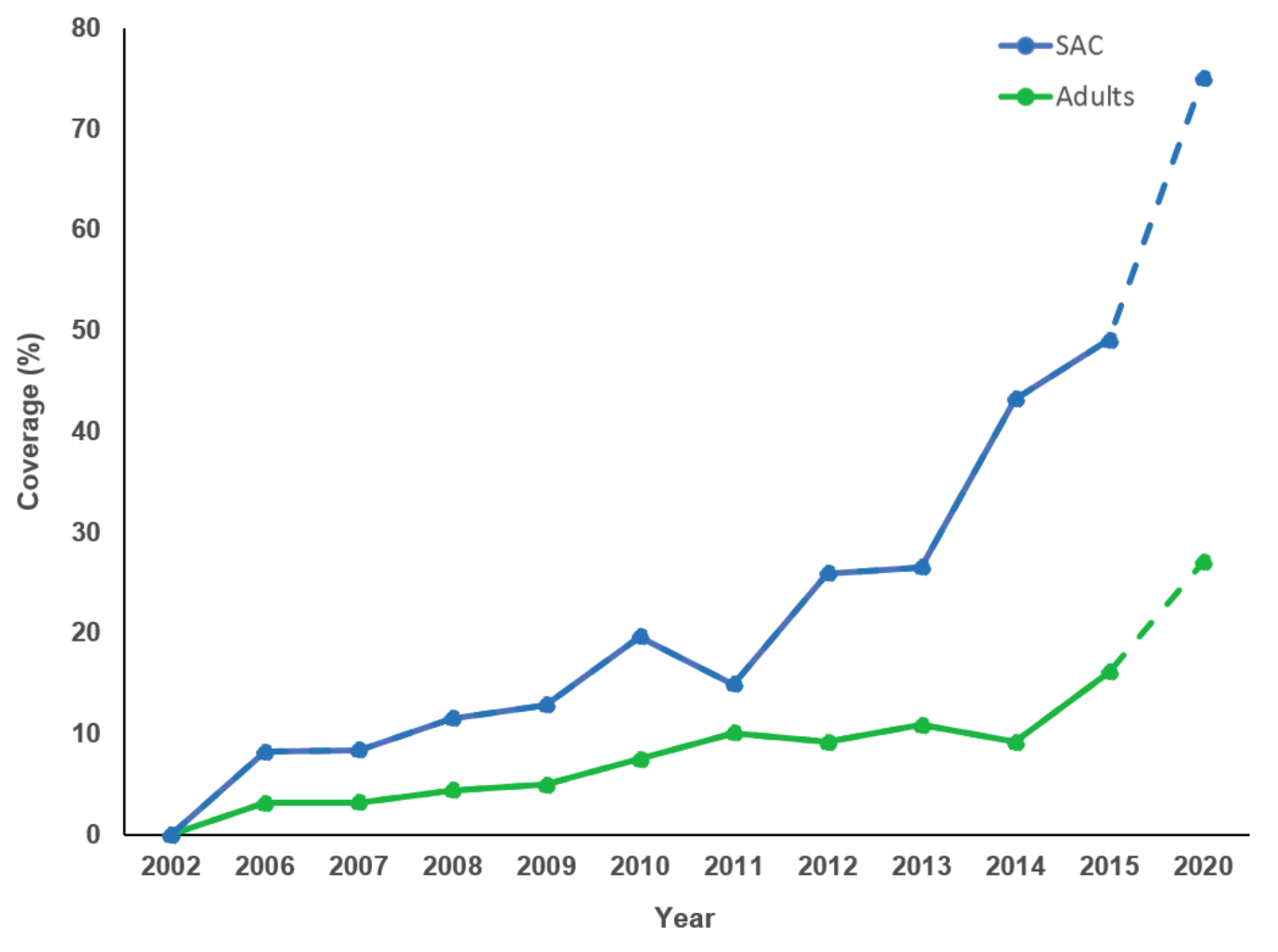
**

**Figure S1:** National coverage in endemic regions as reported by the World Health Organization (WHO) preventive chemotherapy and transmission control (PCT) databank [19].


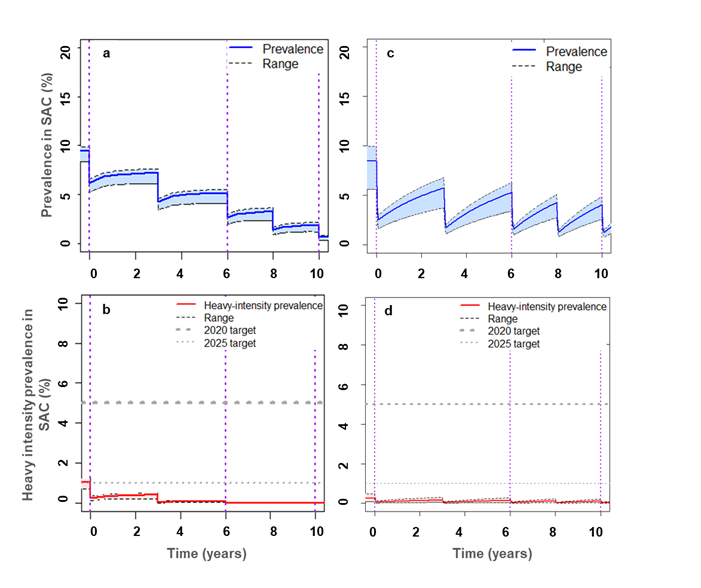


**Figure S2:** ICL (**a**, **b**) and CWRU (**c**, **d**) model scenarios showing prevalence and heavy-intensity infection prevalence in SAC for low baseline prevalence settings. PCT once every 3 years switches to PCT once every 2 years with the elimination goal being reached by year 6 in all simulations. For **a** and **b**: the shaded areas represent the range of R_0_ values (R_0_ = 1.205-1.215). For **c** and **d**: the shaded areas represent the 95% credible interval of uncertainty with the range of ITP values (ITP = 1-5.6). The corresponding CWRU projections for the incidence of infection in the population for low prevalence settings are shown in **Figure S12a**.


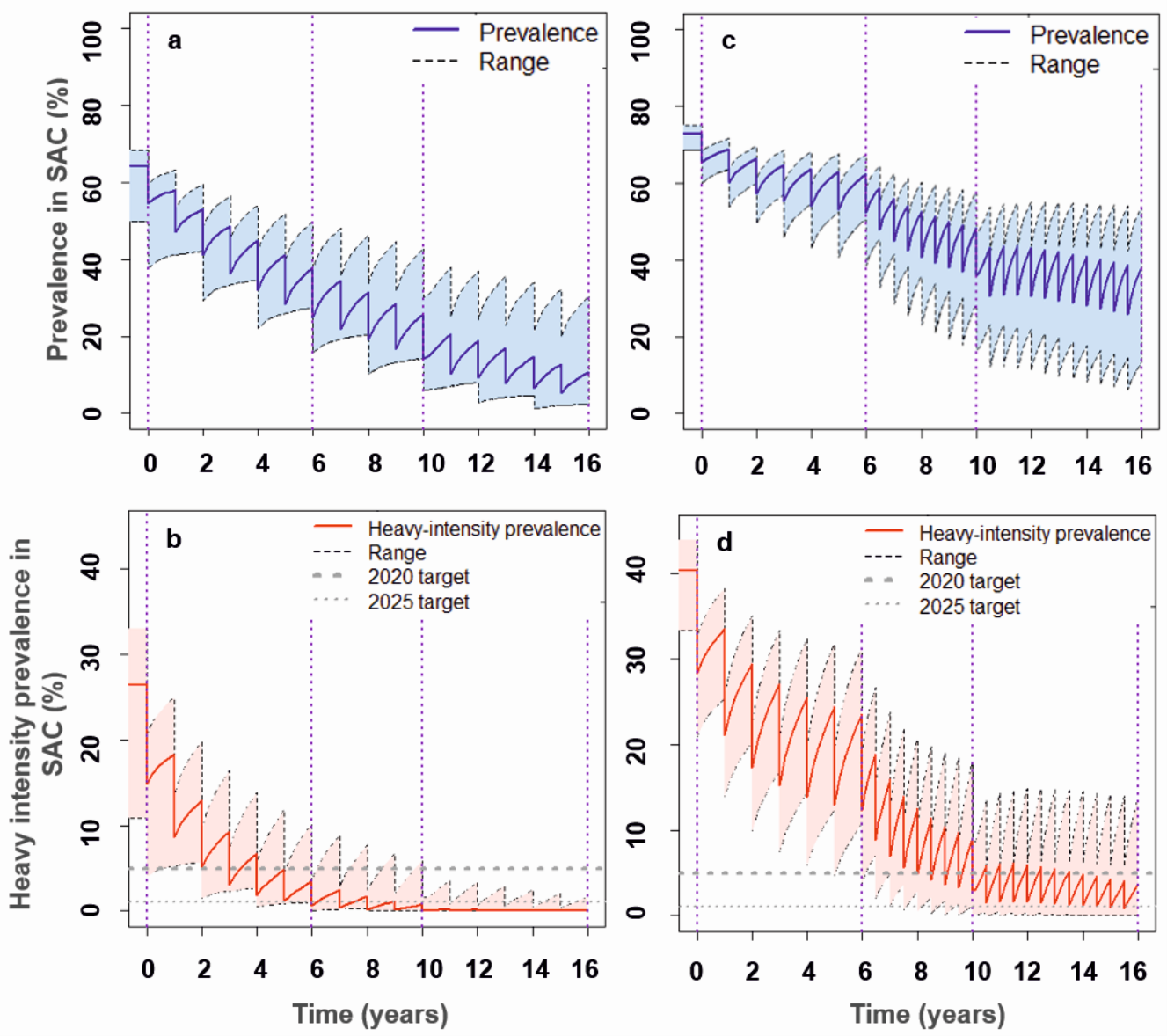


**Figure S3:** ICL model scenarios showing prevalence in SAC and heavy-intensity infection prevalence in SAC for high baseline prevalence settings. Here, treatment has been continued for 6 additional years at year 10 at decision 2 treatment frequency. Improvement varies as in **a** and **b**: R_0_ = 1.24 - 2.4; the morbidity goal is met and the elimination goal may be met. In **c** and **d**: R_0_ = 2.45 - 5.0; the goals may or may not be met depending on the baseline prevalence level.

**
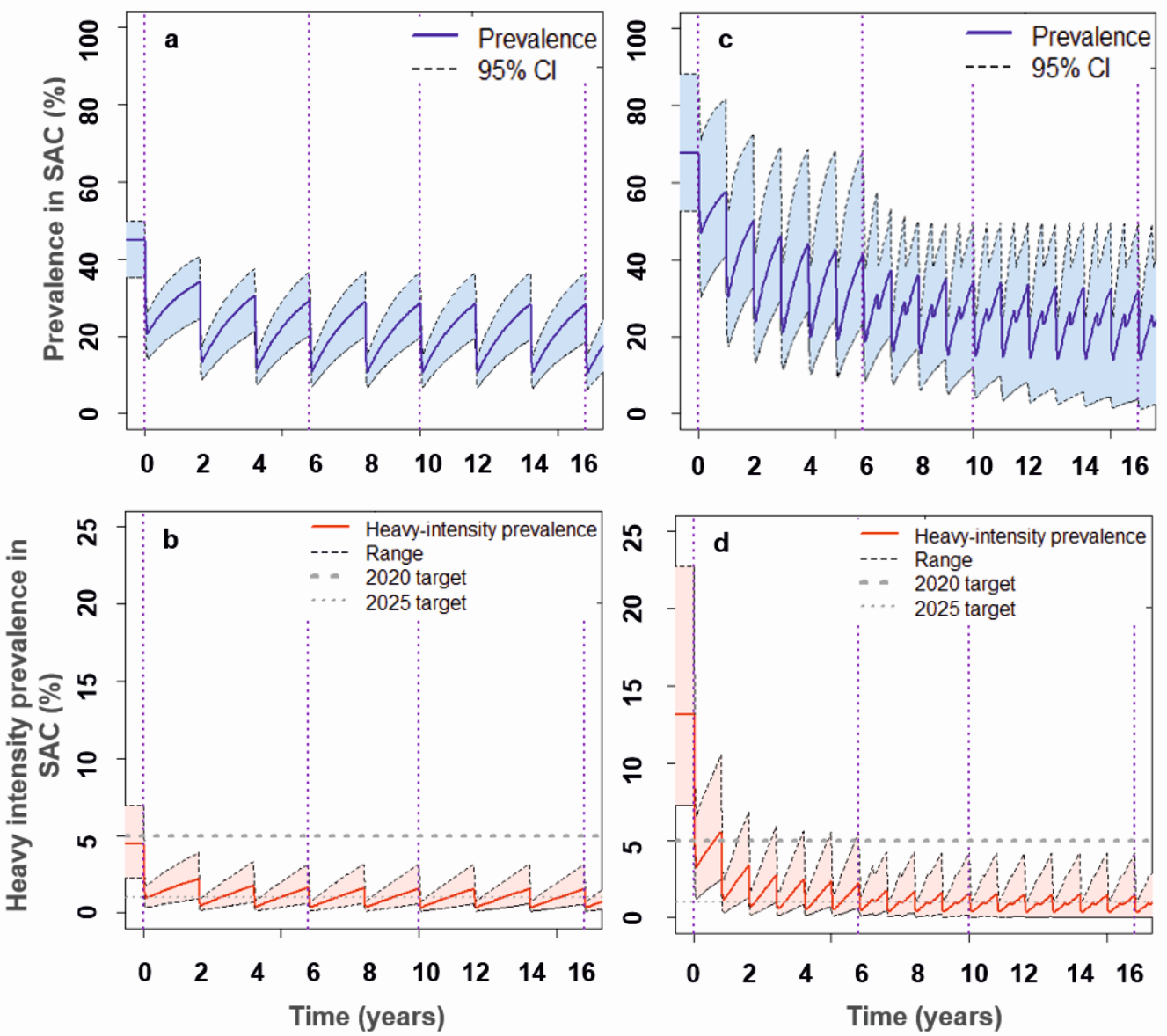
**

**Figure S4:** CWRU model projections for SAC prevalence and heavy-intensity prevalence in **a** and **b**: moderate and **c** and **d**: high baseline prevalence settings. Here, treatment has been continued for 6 additional years at year 10 at decision 2 treatment frequency (from **Figures** **2c, d** and **3c, d**). The probability of elimination as a public health problem in year 16 increased to 21% and 42% for moderate and high prevalence settings, respectively. The shaded areas represent the 95% credible interval of uncertainty in specifying model parameters which translates to multiple simulations with an index of transmission potential (ITP; which is proportional to the magnitude of R_0_) in the range of 1-5.6.

**
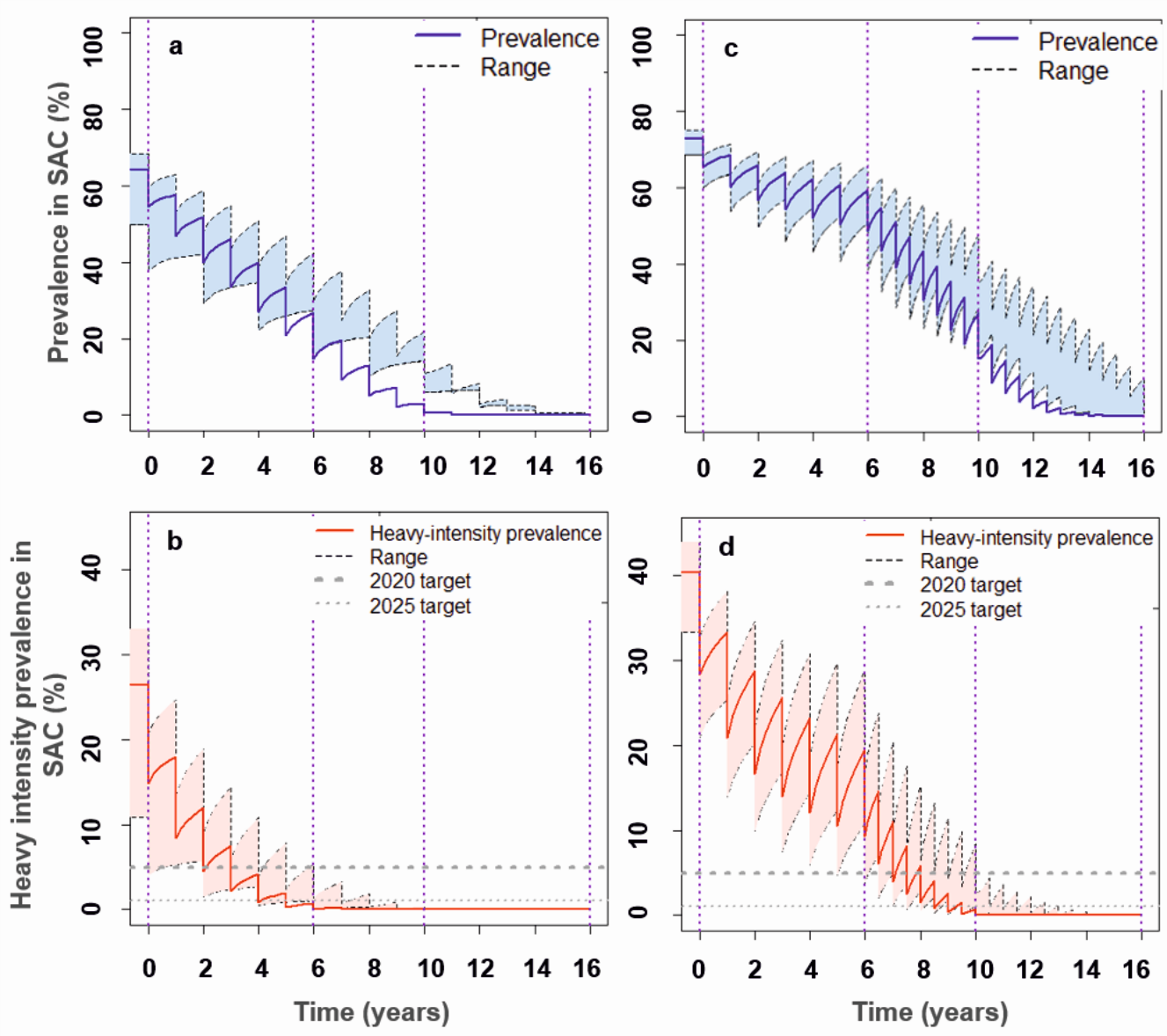
**

**Figure S5:** ICL model scenarios showing prevalence in SAC and heavy-intensity infection prevalence in SAC for high baseline prevalence settings. Here, treatment has been continued for 6 additional years at year 10 at decision 2 treatment frequency and with additional 40% adult coverage. Improvement varies as in **a** and **b**: R_0_ = 1.24 - 2.4 and in **c** and **d**: R_0_ = 2.45 - 5; the morbidity goal or the elimination goal is met.


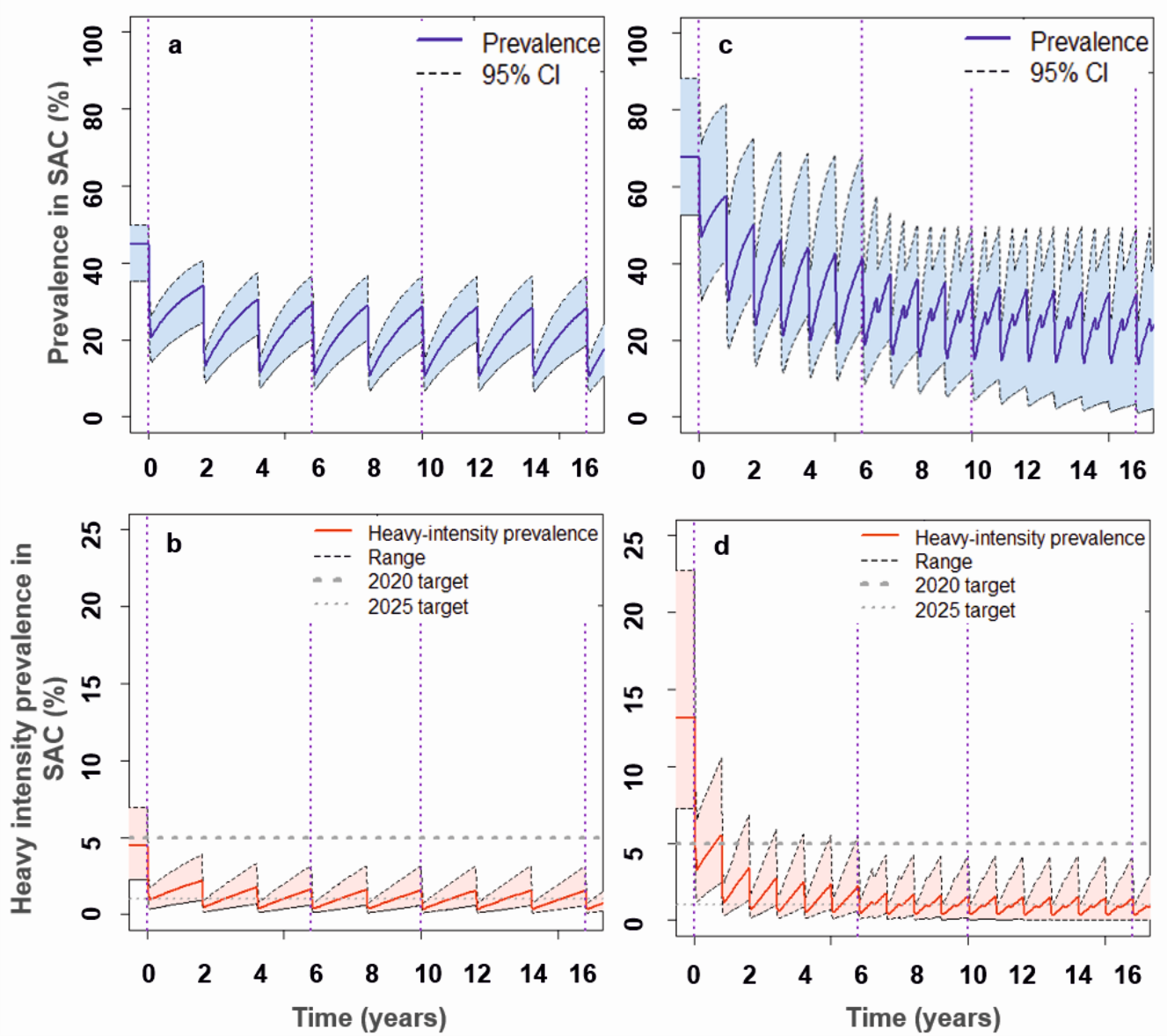


**Figure S6:** CWRU model projections for SAC prevalence and heavy-intensity prevalence in **a** and **b**: moderate and **c** and **d**: high baseline prevalence settings. Here, treatment has been continued for 6 additional years at year 10 at decision 2 treatment frequency and with additional 40% adult coverage (from **Figures 2c, d** and **3c, d**). Although prevalence and heavy-intensity prevalence in year 16 slightly decreased compared to **Figure S4**, the probability of elimination as a public health problem in year 16 remained at 21% and 42% for moderate and high prevalence settings, respectively. The shaded areas represent the 95% credible interval of uncertainty in specifying model parameters which translates to multiple simulations with an index of transmission potential (ITP; which is proportional to the magnitude of R_0_) in the range of 1-5.6.


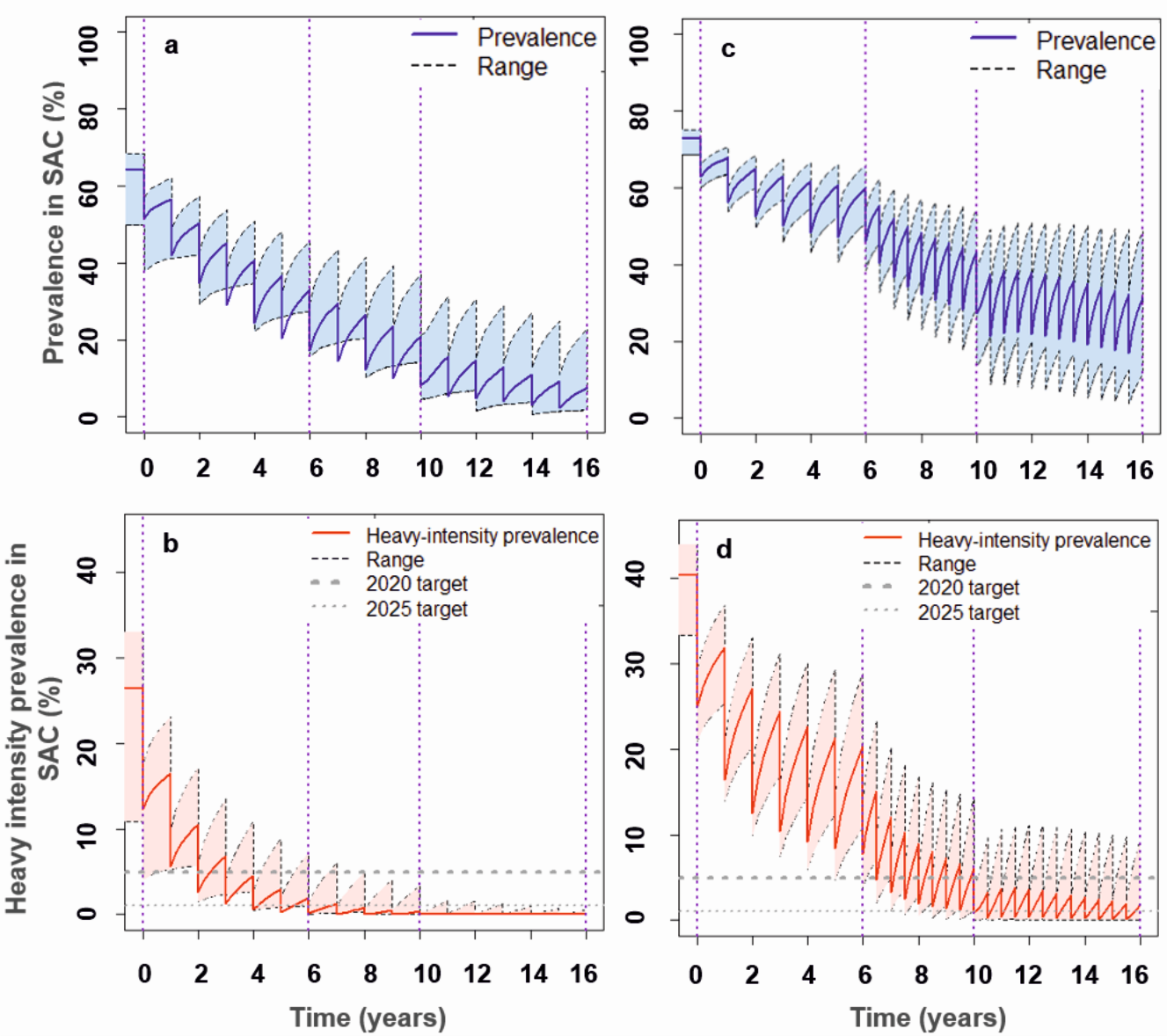


**Figure S7:** ICL model scenarios showing prevalence in SAC and heavy-intensity infection prevalence in SAC for high baseline prevalence settings. Here, treatment has been continued for 6 additional years at year 10 at decision 2 treatment frequency with increased SAC coverage at 85%. Improvement varies as in **a** and **b**: R_0_ = 1.24 - 2.4 and in **c** and **d**: R_0_ = 2.45 - 5; the morbidity goal, elimination goal or no goal is met depending on the baseline prevalence level.


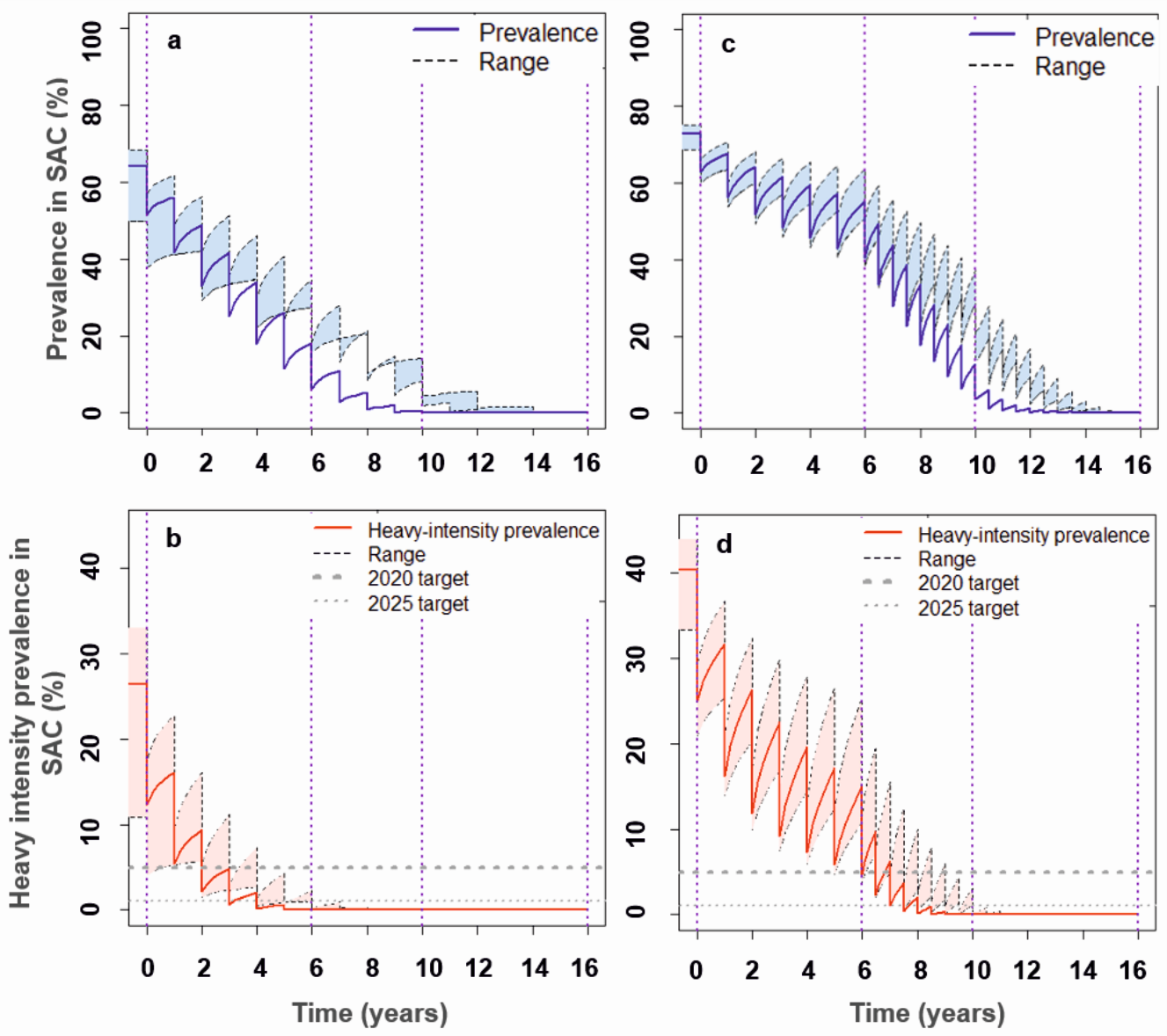


**Figure S8:** ICL model scenarios showing prevalence in SAC and heavy-intensity infection prevalence in SAC for high baseline prevalence settings. Here, treatment has been continued for 6 additional years at year 10 at decision 2 treatment frequency with increased SAC coverage at 85% and inclusion of adult coverage at 40%. Improvement as in **a** and **b**: R_0_ = 1.24 - 2.4 and in **c** and **d**: R_0_ = 2.45 - 5; the elimination goal is likely to be met.


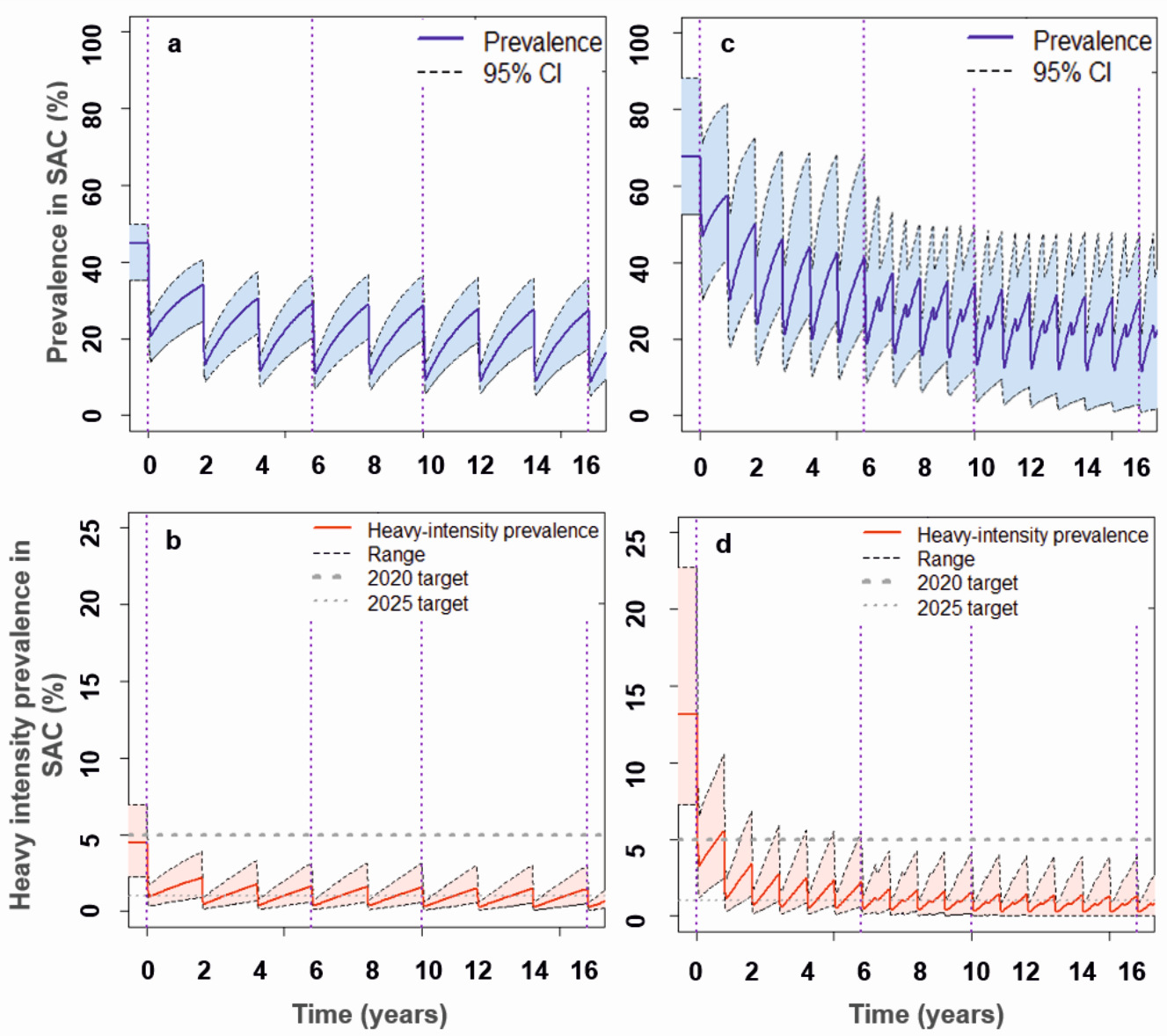


**Figure S9:** CWRU model projections for SAC prevalence and heavy-intensity prevalence in **a** and **b**: moderate and **c** and **d**: high baseline prevalence settings. Here, treatment has been continued for 6 additional years at year 10 at decision 2 treatment frequency with increased SAC coverage at 85% and inclusion of adult coverage at 40% (from **Figures 2c, d** and **3c, d**). The probability of elimination as a public health problem in year 16 increases to 24% and 48% for moderate and high prevalence settings, respectively. The shaded areas represent the 95% credible interval of uncertainty in specifying model parameters which translates to multiple simulations with an index of transmission potential (ITP; which is proportional to the magnitude of R_0_) in the range of 1-5.6.


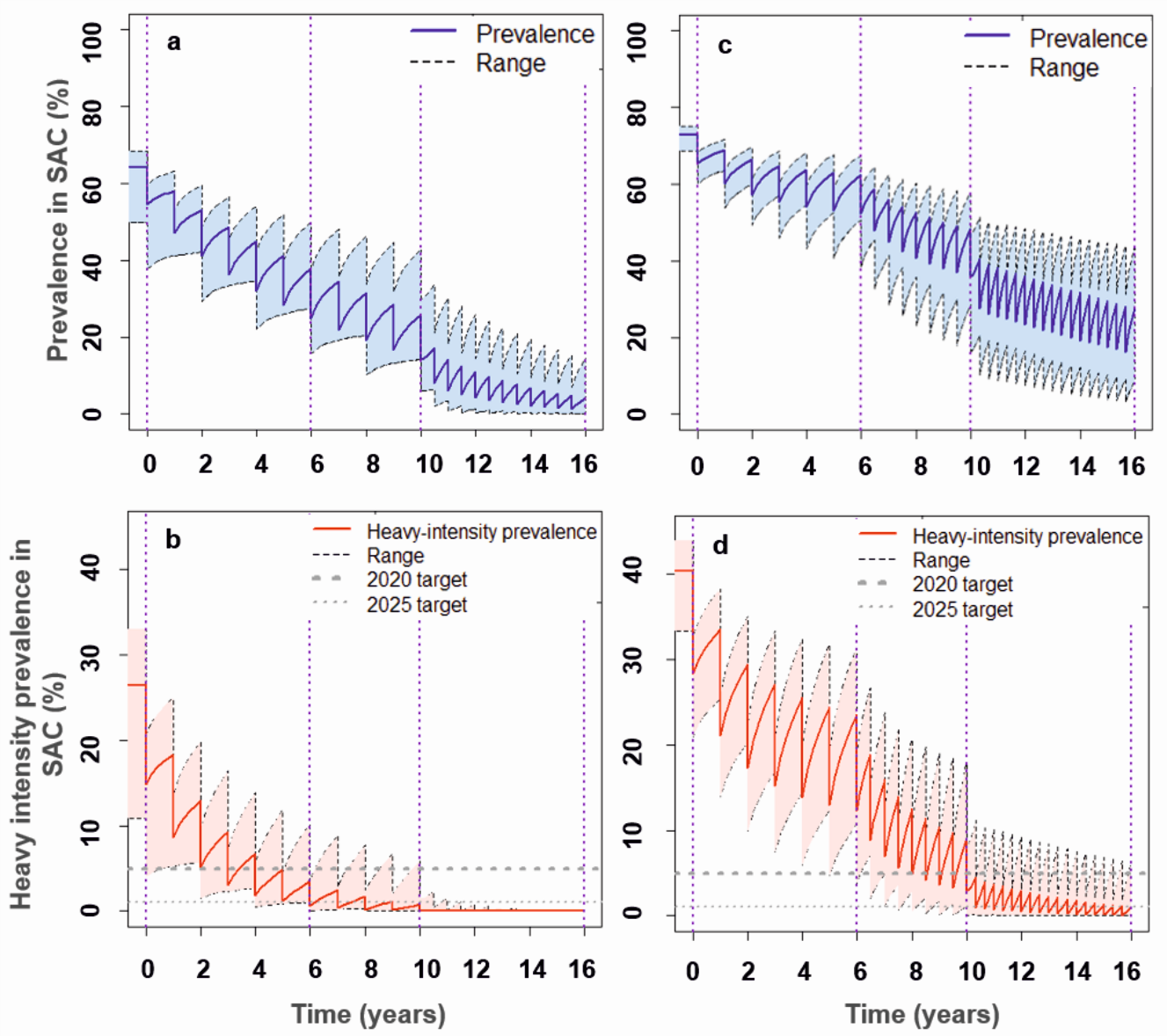


**Figure S10:** ICL model scenarios showing prevalence in SAC and heavy-intensity infection prevalence in SAC for high baseline prevalence settings. Here, treatment has been continued for 6 additional years at year 10 at an increased treatment frequency. Improvement varies as in **a** and **b**: R_0_ = 1.24 - 2.4; treatment frequency is increased from once a year to twice a year which reaches the elimination as a public health problem goal. In **c** and **d**: R_0_ = 2.45 – 5; treatment frequency is increased from twice a year to three times a year which reaches the morbidity goal, elimination goal or no goal depending on the baseline prevalence level.


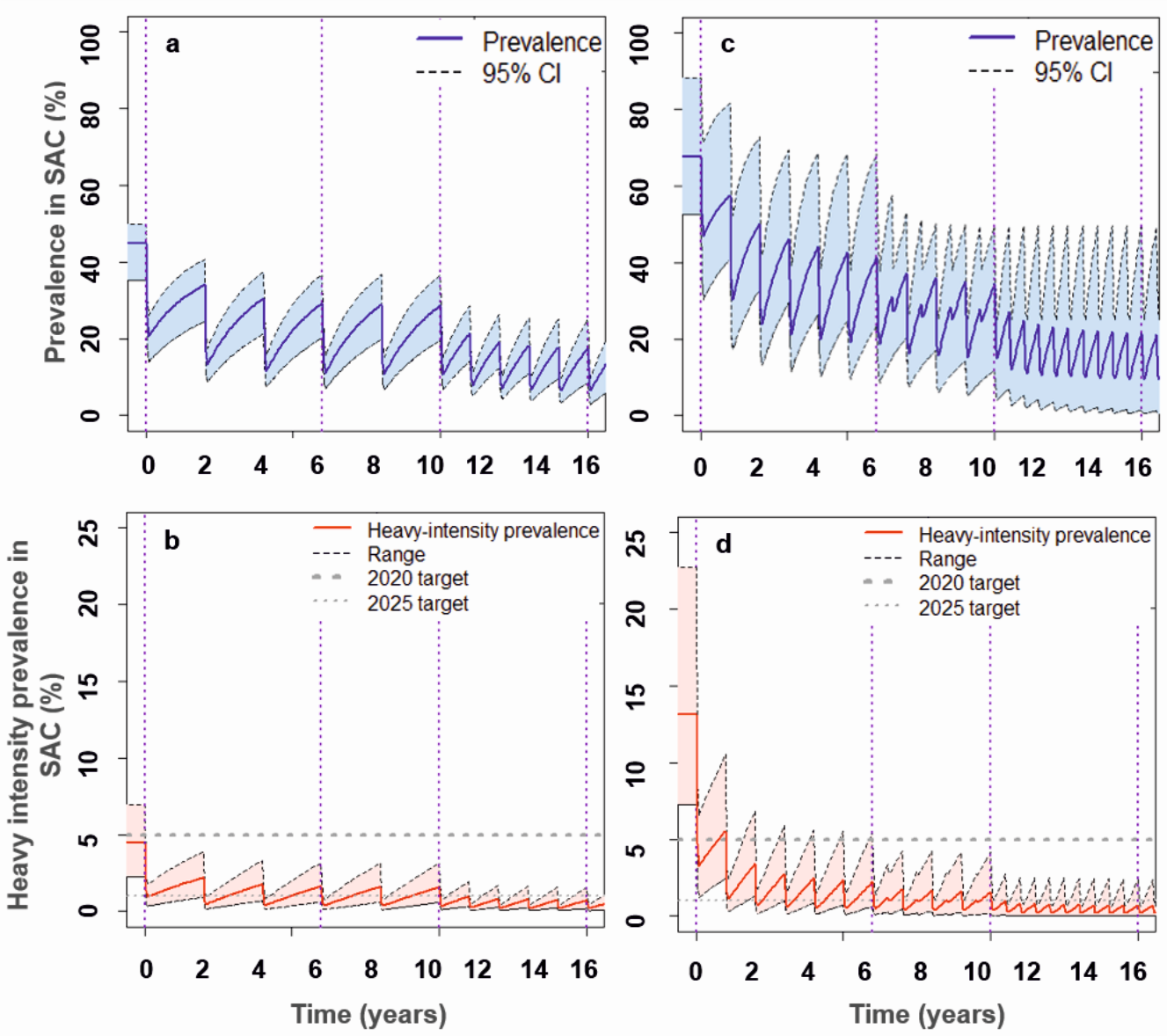


**Figure S11:** CWRU model projections for SAC prevalence and heavy-intensity prevalence in **a** and **b**: moderate and **c** and **d**: high baseline prevalence settings. Here, treatment has been continued for 6 additional years at year 10 at an increased treatment frequency (from **Figures 2c, d** and **3c, d**). In **a** and **b**: moderate baseline prevalence settings; treatment frequency is increased from once every two years to once a year. In **c** and **d**: high baseline prevalence settings; treatment frequency is increased from once a year to twice a year. The probability of elimination as a public health problem at year 16 increases to 81% and 78% for moderate and high prevalence settings, respectively. The shaded areas represent the 95% credible interval of uncertainty in specifying model parameters which translates to multiple simulations with an index of transmission potential (ITP; which is proportional to the magnitude of R_0_) in the range of 1-5.6.

**
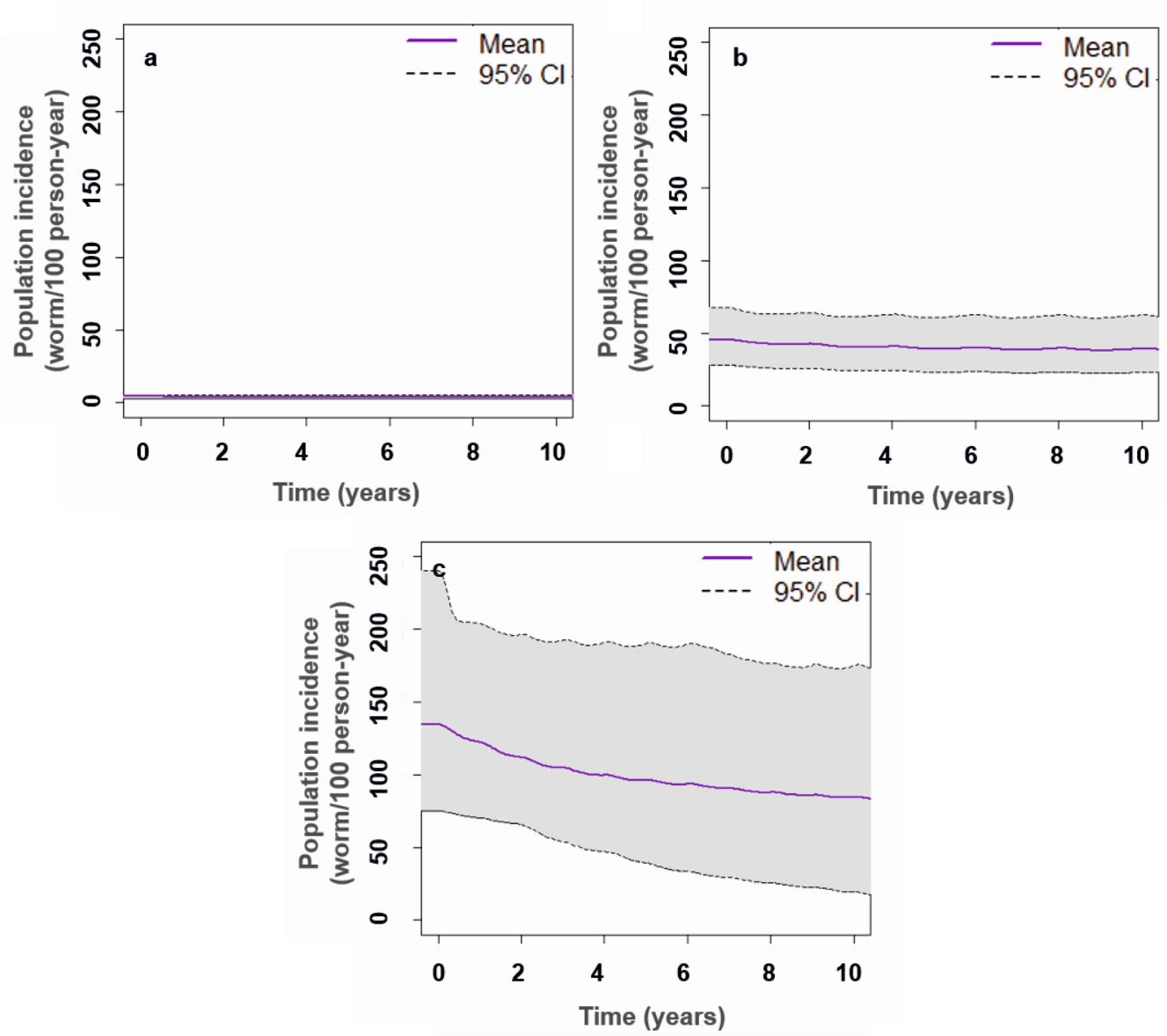
**

**Figure S12:** CWRU model projections on the population incidence in terms of the number of new worms acquired per 100 person-year (corresponding to **Figures S2c-d, 2c-d, 3c-d**) in (**a**) low, (**b**) moderate, and (**c**) high prevalence settings.
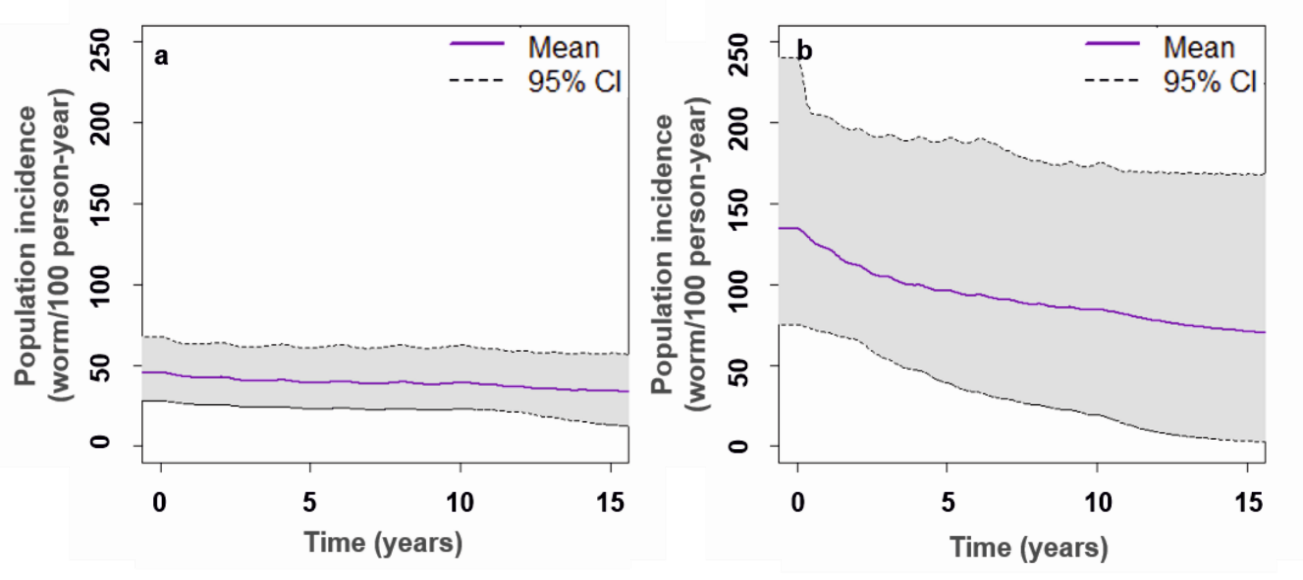


**Figure S13:** CWRU model projections corresponding to the scenarios in **Figure S11** on the population incidence in terms of the number of new worms acquired per 100 person-year in (**a**) moderate, and (**b**) high prevalence settings. Achieving WHO goals in moderate and heavy prevalence settings in **Figure S11** did not translate to achieving eradication of transmission from the population (incidence is still substantially larger than zero).

**References**

1. Truscott JE, Gurarie D, Alsallaq R, et al. A comparison of two mathematical models of the impact of mass drug administration on the transmission and control of schistosomiasis. Epidemics **2017**; 18: 29-37.

2. Anderson RM, Turner HC, Farrell SH, Truscott JE. Studies of the transmission dynamics, mathematical model development and the control of schistosome parasites by mass drug administration in human communities. Adv Parasitol **2016**; 94: 199-246.

3. Anderson RM, Turner HC, Farrell SH, Yang J, Truscott JE. What is required in terms of mass drug administration to interrupt the transmission of schistosome parasites in regions of endemic infection? Parasit Vectors **2015**; 8: 553.

4. Gurarie D, Yoon N, Li E, et al. Modelling control of Schistosoma haematobium infection: predictions of the long-term impact of mass drug administration in Africa. Parasit Vectors **2015**; 8: 529.

5. Alsallaq RA, Gurarie D, Ndeffo Mbah M, Galvani A, King C. Quantitative assessment of the impact of partially protective anti-schistosomiasis vaccines. PLoS Negl Trop Dis **2017**; 11(4): e0005544.

6. Anderson RM, M. MR. Infectious diseases of humans: dynamics and control: Oxford University Press, **1991**.

7. De Vlas SJ, Gryseels B, Van Oortmarssen GJ, Polderman AM, Habbema JD. A model for variations in single and repeated egg counts in Schistosoma mansoni infections. Parasitology **1992**; 104 ( Pt 3): 451-60.

8. Anderson RM, Crombie J. Experimental studies of age-prevalence curves for Schistosoma mansoni infections in populations of Biomphalaria glabrata. Parasitology **1984**; 89 ( Pt 1): 79-105.

9. Carter NP, Anderson RM, Wilson RA. Transmission of Schistosoma mansoni from man to snail: laboratory studies on the influence of snail and miracidial densities on transmission success. Parasitology **1982**; 85 (Pt 2): 361-72.

10. NTD Modelling Consortium. Schistosomiasis Mansoni. Available at: <http://www.ntdmodelling.org/diseases/schistosomiasis-mansoni>.

11. Ezeamama AE, He CL, Shen Y, et al. Gaining and sustaining schistosomiasis control: study protocol and baseline data prior to different treatment strategies in five African countries. BMC Infect Dis **2016**; 16: 229.

12. de Vlas SJ, Nagelkerke NJ, Habbema JD, van Oortmarssen GJ. Statistical models for estimating prevalence and incidence of parasitic diseases. Stat Methods Med Res **1993**; 2(1): 3-21.

13. Anderson RM, May RM. Population dynamics of human helminth infections: control by chemotherapy. Nature **1982**; 297(5867): 557-63.

14. Kostitzin VA. Symbiosis, parasitism and evolution. In: Scudo FM, Ziegler JR. The Golden Age of Theoretical Ecology: 1923-1940, **1934**:369-408.

15. Chan MS, Guyatt HL, Bundy DA, Booth M, Fulford AJ, Medley GF. The development of an age structured model for schistosomiasis transmission dynamics and control and its validation for Schistosoma mansoni. Epidemiol Infect **1995**; 115(2): 325-44.

16. Fulford AJ, Butterworth AE, Ouma JH, Sturrock RF. A statistical approach to schistosome population dynamics and estimation of the life-span of *Schistosoma mansoni* in man. Parasitology **1995**; 110 ( Pt 3): 307-16.

17. Zwang J, Olliaro PL. Clinical efficacy and tolerability of praziquantel for intestinal and urinary schistosomiasis-a meta-analysis of comparative and non-comparative clinical trials. PLoS Negl Trop Dis **2014**; 8(11): e3286.

18. Committee WHOE. Prevention and control of schistosomiasis and soil-transmitted helminthiasis. World Health Organ Tech Rep Ser **2002**; 912: i-vi, 1-57, back cover.

19. World Health Organization. PCT databank: Schistosomiasis. Available at: <http://www.who.int/neglected_diseases/preventive_chemotherapy/sch/en/>.
